# CAPRIN2 upregulation by LINC00941 promotes nasopharyngeal carcinoma ferroptosis resistance and metastatic colonization through HMGCR

**DOI:** 10.3389/fonc.2022.931749

**Published:** 2022-10-06

**Authors:** Lin Qiu, Rui Zhou, Ling Zhou, Shiping Yang, Jiangxue Wu

**Affiliations:** ^1^ State Key Laboratory of Oncology in South China, Collaborative Innovation Center for Cancer Medicine, Sun Yat-sen University Cancer Center, Guangzhou, China; ^2^ Guangzhou Women and Children’s Medical Center, Department of Hematology and Oncology, Guangzhou Medical University, Guangzhou, China; ^3^ Department of General Surgery, The Third Affiliated Hospital of Southern Medical University, Guangzhou, China; ^4^ Department of Radiation Oncology, Hainan Affiliated Hospital of Hainan Medical University, Haikou, China

**Keywords:** CAPRIN2, ferroptosis, metastasis, nasopharyngeal carcinoma, survival

## Abstract

Distant metastasis is the main cause of death in nasopharyngeal carcinoma (NPC) patients. There is an urgent need to reveal the underlying mechanism of NPC metastasis and identify novel therapeutic targets. The ferroptosis resistance and survival ability of extracellular matrix (ECM)-detached tumor cells are important factors in determining the success of distant metastasis. In this study, we found that CAPRIN2 contributes to the ferroptosis resistance and survival of ECM-detached NPC cells. Moreover, CAPRIN2 serves as a positive regulator of NPC cell migration and invasion. HMGCR, the key metabolic enzyme of the mevalonate pathway, was identified as the key downstream molecule of CAPRIN2, which mediates its regulation of ferroptosis, survival, migration and invasion of NPC cells. Lung colonization experiments showed that downregulation of the CAPRIN2/HMGCR axis resulted in reduced lung metastasis of NPC cells. Erastin treatment inhibited the ability of NPC cells to colonize the lungs, which was further enhanced by CAPRIN2/HMGCR axis downregulation. Regulated by upstream LINC00941, CAPRIN2 is abnormally activated in NPC, and its high expression is associated with a poor prognosis. In conclusion, CAPRIN2 is a molecular marker of a poor prognosis in NPC, and the LINC00941/CAPRIN2/HMGCR axis provides a new target for the treatment of NPC metastasis and ferroptosis resistance.

## Introduction

Nasopharyngeal carcinoma (NPC) is an Epstein-Barr virus (EBV)-associated tumor, and the main pathological type is undifferentiated carcinoma. It is characterized by high aggressiveness and metastatic potential (1–6). At the time of onset, 5% to 11% of patients have distal metastases (1). During the course of treatment, 50% to 60% of patients develop distal metastasis (1). Due to advances in radiotherapy techniques and increases in the accuracy of disease staging, the overall prognosis of NPC has improved significantly over the past three decades (1–5). However, distal metastasis is still the main cause of death in NPC patients (1–5). Therefore, there is an urgent need to explore the potential mechanism of NPC metastasis and identify specific biomarkers.

Ferroptosis is a type of iron-dependent cell death characterized by lipid peroxidation mediated by reactive oxygen species (ROS) (7–18). Until now, the role of ferroptosis in the complex process of tumor cell metastasis has remained poorly understood. To successfully metastasize from the primary site to the distal organ, tumor cells must overcome several obstacles, including long-term survival after extracellular matrix (ECM) detachment and distal organ colonization (19–28). Only tumor cells that are resistant to ECM detachment-induced cell death and can adapt to the distal organ microenvironment are likely to survive and successfully colonize to form metastases (23–27). It is well known that ECM-detached tumor cells undergo anoikis, a type of caspase-dependent programmed cell death (19–21). However, as the understanding of the complex changes in cells induced by ECM detachment has deepened, studies have shown that resistance to anoikis alone is not sufficient to maintain long-term cell survival after ECM detachment, suggesting that other modes of death may be involved (19–22). In 2017, Brown et al. reported that ECM detachment is an important trigger factor for ferroptosis (29). ECM detachment results in a dramatic increase in ROS and leads to the ferroptosis of breast cancer cells (29). In addition, metastatic tumor cells in the lung are exposed to a high oxygen microenvironment. Only the cells that can successfully resist the oxidative damage and ferroptosis induced by high oxygen levels can colonize and form clones in the new microenvironment (30). To date, the role of ferroptosis in NPC metastasis has not been studied. The mechanism by which ECM-detached NPC cells resist ferroptosis to maintain survival remains unknown.

Caprin family member 2 (CAPRIN2) is an RNA-binding protein (RBP) that functions in the central osmotic defense response and eye development (31–33). The function of CAPRIN2 in tumors is still poorly understood. Jia et al. identified gain-of-function CAPRIN2 mutations (R968H/S969C) in hepatoblastoma that promote the growth of hepatoblastoma cells (34). In addition, upregulation of CAPRIN2 was found to promote oral squamous cell carcinoma (OSCC) by activating the canonical WNT/β-catenin signaling pathway (35). Thus far, the role of CAPRIN2 in NPC remains unknown. Moreover, the functions of CAPRIN2 in tumor ferroptosis have not been reported.

Here, we investigated the potential role of CAPRIN2 in NPC ferroptosis and metastasis. Our results indicated that CAPRIN2 acts as a protector against NPC cell ferroptosis. Moreover, the upregulation of CAPRIN2 promotes the survival, migration and invasion of NPC cells. The 3-hydroxy-3-methylglutaryl-CoA reductase (HMG-CoA reductase, HMGCR) functions as the key downstream molecule of CAPRIN2. CAPRIN2/HMGCR might be novel therapeutic targets for the development of treatments for NPC.

## Materials and methods

### Cell cultures

The NPC cell lines involved in the study included the EBV-negative cell lines 5-8F (poorly differentiated), 6-10B (poorly differentiated) and HK-1 (well differentiated); the EBV-positive cell line C666-1 (undifferentiated); and the immortalized normal nasopharyngeal epithelial cell line NP69. C666-1, HK-1 and NP69 cells were kindly provided by Dr. Saiwah Tsao (University of Hong Kong, Hong Kong, P.R. China), and the 5-8F and 6-10B cell lines were maintained by our laboratory. Cells were maintained in DMEM or RPMI-1640 medium supplemented with 10% fetal bovine serum, 100 units/mL penicillin, and 100 μg/mL streptomycin at 37°C in a 5% humidified CO_2_ atmosphere. The indicated cell lines were routinely detected and ensured to be mycoplasma-free using a PCR-based method.

### Reagents and antibodies

The ferroptosis inducer and cell death inhibitor were all obtained from Selleck (Shanghai, China). The ferroptosis activator used was erastin. In order to facilitate understanding the effects of CAPRIN2 on ferroptosis, we chose the erastin doses with a growth inhibition rate of 30-40% of the control group. Otherwise, if the erastin dose is too low, the growth inhibition effect will be too weak to study the effect of CAPRIN2 on ferroptosis resistance. Similarly, it is not suitable to study the effect of knockdown of CAPRIN2 on ferroptosis if the erastin dose is too high. The ferroptosis inhibitor used was ferrostatin-1. Ferrostatin-1 is a lipophilic antioxidant that acts through a free radical trapping mechanism that can prevent the accumulation of lipid peroxidation induced by erastin, thereby inhibiting ferroptosis (9). MVA was obtained from Sigma (Shanghai, China). Primary antibodies against CAPRIN2 (NBP1-88318, Novus), HMGCR (sc-271595, Santa Cruz) and β-actin (66009-1-Ig, Proteintech, Wuhan, China) were commercially obtained.

### Cells transfection

The siRNAs applied in the study were all products of Santa Cruz (Shanghai, China) and are listed as follows: CAPRIN2 siRNA, HMGCR siRNA and negative control siRNA. The pcDNA3.1 vector carrying the cDNA sequence of CAPRIN2 or HMGCR was constructed by Generay Biotech (Shanghai, China). The cDNA sequence of CAPRIN2 or HMGCR was also subcloned into the lentivirus vector pHBLV-CMV-MCS-EF1-NEO. The obtained plasmids were named pHBLV-CAPRIN2 or pHBLV-HMGCR. Lipofectamine 2000 (Thermo Fisher, Shanghai, China) was used for transient transfection of the indicated siRNA or plasmid.

### Stable cell line construction

To construct stable cell lines with knockdown of CAPRIN2 or HMGCR, lentiviruses carrying CAPRIN2 shRNA, HMGCR shRNA or scramble shRNA were purchased from Santa Cruz (CA, USA) and used to infect the indicated NPC cell lines for 48 h. The sequence of human LINC00941 shRNA was 5′- GAGACAGTTGATAGCCAAA -3′ (36), and the constructs were cloned into pHBLV-U6-MCS-PGK-PURO, named pHBLV-shLINC00941. pHBLV-shLINC00941 was transfected into 293T cells along with the corresponding packaging vector PMD2.G and pSPAX2. Cell supernatants were harvested at 48 h after transfection and used to infect the indicated NPC cells. The stably infected cells above were selected with puromycin (2 µg/mL) for two weeks.

To stably overexpress CAPRIN2 or HMGCR, the pHBLV-CAPRIN2 or pHBLV-HMGCR plasmid was transfected into 293T cells along with the corresponding packaging vector PMD2.G and pSPAX2, respectively. Cell supernatants were harvested at 48 h after transfection and were subsequently used to infect the indicated NPC cells. Stably infected cells were selected with G418 (0.5 mg/mL) for two weeks.

For the above stable cell lines, the overexpression or knockdown efficiency of the indicated genes or lncRNA was validated by qRT-PCR and/or Western blot analysis.

### RNA isolation and real-time reverse transcription PCR

Total RNA was extracted from NPC cell lines or tissues using TRIzol reagent (Invitrogen, Carlsbad, CA, USA) according to the manufacturer’s instructions. A reverse transcriptase system (Promega, Madison, WI, USA) was applied to synthesize cDNA, and real-time PCR was performed using SYBR green master mix (Invitrogen, CA, USA). The relative expression of target genes was normalized to that of β-actin, and quantified by the 2^-ΔΔCt^ method. All reactions were performed in triplicate in three independent experiments. The primers used for the amplification of the indicated genes or lncRNA are listed in **Supplementary Table S1**.

### Western blotting

Cells were collected and total protein was extracted in a lysis buffer containing protease inhibitors (Thermo Fisher, Shanghai, China). Western blot analysis was performed as previously described (37).

### Cell viability analysis

Cell viability was evaluated using the AlamarBlue Cell Viability Assay Kit (Thermo Fisher, IL, USA) according to the manufacturer’s instructions. The growth inhibition rate presented reflects the growth inhibitory effect of erastin treatment on cells compared to the cells without erastin treatment. The growth inhibitory rate is obtained as follows: growth inhibition rate (%)=100%-(viability of the indicated group with erastin treatment)/(viability of the control group without erastin treatment) × 100%. For the control group without erastin treatment, the inhibition rate is 0.

### Malondialdehyde assay

The Lipid Peroxidation (MDA) Assay Kit was purchased from Abcam (MA, USA). The MDA content was tested according to the manufacturer’s instructions.

### Reduced glutathione assay

The Reduced Glutathione Assay Reagent Kit purchased from Solarbio (Beijing, China) was used to measure the cellular GSH concentration.

### Survival analysis of NPC cells under ECM-detached culture conditions

The cells were inoculated on a 24-well ultralow attachment plate with the optimal cell density (500 cells/well for 5-8F; 1000 cells/well for C666-1), and serum-free DMEM/F-12 culture medium (20 ng/ml bFGF, 20 ng/ml EGF, and 20 ng/ml insulin) was used to study the survival of ECM-detached NPC cells. Fresh serum-free DMEM/F12 medium containing growth factors was supplemented every other day. The culture medium and reagents were products of Cell Signaling (Shanghai, China) and Thermo Fisher (IL, USA). After 72 h of culture, the viability of NPC cells was assessed by Alamar Blue assay.

### Transwell migration and invasion assay

Transwell assays were performed using 24-well Transwell chambers (8-μm pore; BD Falcon) to evaluate the migration or invasion properties of the indicated cells. For the 5-8F cells, 1.5×10^4^ cells in serum-free 1640 medium were added into the upper sides of the insert membrane with or without Matrigel, while the bottom chamber was supplemented with 1640 medium containing 10% FBS. After 16 h of incubation, the NPC cells that did not migrate or invade the membrane were scraped off, and cells on the bottom of the membrane were fixed with crystal violet. For the C666-1 cell line, 5×10^4^ cells suspended in 1640 medium containing 1% FBS were added to the upper sides of the inserts coated with 20 μg/mL fibronectin (Cell Signaling, Shanghai, China). Fibronectin was applied as a chemoattractant at a final concentration of 50 μg/mL. After 24 h, cells on the bottom of the membrane were stained. The cells in five independent fields were counted under a microscope at a magnification of 10×. The experiments above were performed in triplicate.

### *In vivo* lung colonization models

The animal experiments in the study were conducted in accordance with the NIH animal use guidelines and were approved by the Sun Yat-sen University Institutional Animal Care and Use Committee. Nude female BALB/c mice (4 weeks) were purchased from the SLACCAS Experimental Animals Co., Ltd. (Shanghai, China), and were maintained under specific pathogen-free conditions.

For the lung metastasis model, mice were randomly assigned to four groups (n=6), and a total of 1×10^6^ of the indicated cells in 100 μL PBS were injected into the tail vein of the mice. Three weeks after the injection, the mice were euthanized, and the lungs were harvested and stained with hematoxylin-eosin for pathologic analysis. Metastatic nodules were observed using a microscope.

To further explore the role of the CAPRIN2/HMGCR axis in the lung colonization capacity of NPC cells treated with erastin, the mice were divided into four groups (n=6) and injected with 1×10^6^ of the indicated stable cell lines into the tail veins. From day 0 of cell injection, the mice were intraperitoneally administered erastin (40 mg/kg) or vehicle as control twice every other day. After 21 days of cell injections, the mice were sacrificed and the lungs were harvested for histological analysis. The number of metastatic nodules was calculated under a microscope.

### Patient tumor samples and immunohistochemistry

NPC tumor tissues were collected from 104 patients histologically diagnosed with NPC at Sun Yat-sen University Cancer Center (SYSUCC) between 2007 and 2012. The inflammatory nasopharynx tissues were collected *via* outpatient biopsy. The TMA was generated from formalin-fixed, paraffin-embedded NPC tissues. Informed consent forms were obtained, and the study was approved by the Institutional Research Ethics Committee of SYSUCC. No patient received treatment before biopsy. The CAPRIN2 protein level was assessed according to immunohistochemical staining intensity. Immunohistochemistry was performed as previously described with the appropriate modifications (38). A polyclonal anti-CAPRIN2 antibody was obtained from Novus (1:500, NBP1-88318). The degree of staining in the sections was observed and scored independently by two observers who were not informed of the clinical data of the patients evaluated the staining intensity. The expression intensity was classified as negative = 0, weak = 1, moderate = 2, or strong = 3. The final H score was calculated based on multiplying the intensity score by the percentage of the staining area. A receiver operating characteristic (ROC) curve analysis was applied to determine a cutoff value for CAPRIN2 low expression and high expression. The sensitivity and specificity for the H score was plotted, thus generating a ROC curve. The score that was closest to the point with both the maximum sensitivity and specificity was selected as the cut-off value.

### Statistical analysis

All statistical analyses were completed with SPSS statistical software (version 17.0, SPSS Inc., Chicago, IL, USA). Student’s *t-test* was applied to determine the difference between two groups. Survival curves were plotted using the *Kaplan-Meier* method and compared using the *log-rank test*. A two-tailed *chi-square test* was used to analyze the correlation between CAPRIN2 expression and the clinicopathological characteristics. Univariate and multivariate survival analyses were conducted using Cox’s proportional hazard model. The relationship between two independent variables was evaluated by the *Pearson* correlation coefficients method. p < 0.05 was considered to indicate a statistically significant difference.

## Results

### CAPRIN2 promotes the ferroptosis resistance and survival of ECM-detached NPC cells

The ability to survive the stress of ECM detachment is one of the important factors to determine the successful metastasis of tumor cells. To evaluate the biological effects of CAPRIN2 on the ferroptosis resistance and survival of ECM-detached NPC cells both *in vitro* and *in vivo*, we constructed NPC cell lines with stable CAPRIN2 knockdown or overexpression.

First, we determined the endogenous expression levels of CAPRIN2 in NPC cells by qRT-PCR and Western blotting. The results showed that CAPRIN2 was consistently highly expressed in all NPC cell lines assessed compared to the nasopharyngeal epithelial cell line NP69 (**Supplementary Figures S1A, B**). Then, we selected 5-8F (a poorly differentiated NPC cell line with high metastatic capacity) and C666-1 (an EBV-positive undifferentiated NPC cell line) cells to construct NPC cell lines with stable CAPRIN2 knockdown and overexpression, respectively (**Supplementary Figure S2**).

**Figure 1:**
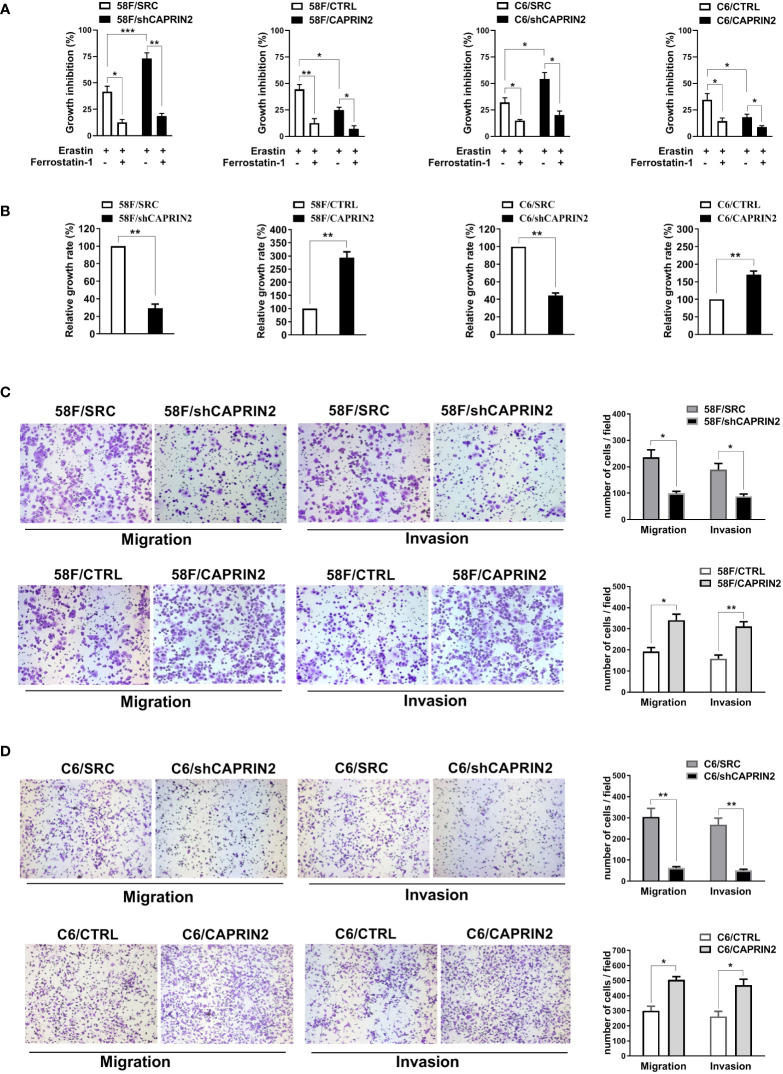
Effects of CAPRIN2 on the ferroptosis and survival of ECM-detached NPC cells and the migration and invasion of NPC cells. **(A)** Viability assay of ECM-detached NPC cell lines treated with erastin (5 μM) and/or ferrostatin-1 (1 μM) for 24 h **(B)** Viability assay of the indicated stable 5-8F or C666-1 cell lines cultured under ECM detachment conditions for 72 h For **(A, B)**, the experiments were repeated three times, and the data are shown as the mean ± SEM. * p < 0.05, ** p < 0.01, *** p < 0.001. **(C, D)** Migration and invasion assays of the indicated stable 5-8F **(C)** and C666-1 **(D)** cell lines. These assays were conducted in triplicate. Representative images are displayed. The data are presented as the mean ± SD. * p < 0.05, ** p < 0.01, *** p < 0.001.

Next, stable NPC cell lines were cultured using ultralow attachment plates and treated with erastin to investigate the ferroptosis of ECM-detached NPC cells. Twenty-four hours later, the viability of cells was evaluated by Alamar Blue Assay. The results showed that erastin inhibited the growth of NPC cells, and this effect was attenuated by ferrostatin-1 (ferroptosis inhibitor) (**Figure 1A**). The stable knockdown of CAPRIN2 in NPC cells enhanced erastin induced ferroptosis, while the stable overexpression of CAPRIN2 promoted ferroptosis resistance in NPC cells (**Figure 1A**).

The survival of cells under ECM-detached culture conditions was also evaluated by Alamar Blue Assay. As shown in **Figure 1B**, in 5-8F or C666-1 cells, knockdown CAPRIN2 inhibited the survival of ECM-detached cells, whereas overexpression of CAPRIN2 promoted cell survival.

### CAPRIN2 promotes the migration and invasion of NPC cells *in vitro*

Migration and invasion are also important factors affecting NPC cell metastasis. We examined the migration and invasion ability of NPC cells after knockdown or overexpression of CAPRIN2. The results of the Transwell assay indicated that downregulation of CAPRIN2 reduced the migration and invasion capability of 5-8F cells, while overexpression of CAPRIN2 significantly promoted cell migration and invasion (**Figure 1C**). Consistent results were also obtained in C666-1 cells (**Figure 1D**).

### CAPRIN2 activates HMGCR, a key enzyme in the mevalonate pathway

The main ferroptosis suppression systems include the cyst(e)ine/GSH/GPX4 axis, the NAD(P)H/FSP1/CoQ10 system and the GCH1/BH4/DHFR system (9). In the 5-8F cell line with stable CAPRIN2 knockdown, we evaluated key molecules involved in the above inhibition systems. CAPRIN2 significantly regulated HMGCR, and consistent regulation was also detected in C666-1 cells (**Supplementary Figure S3A**). However, no significant change in the expression level of the remaining ferroptosis regulatory molecules was detected (**Supplementary Figure S3A**). In addition, we examined key molecules related to iron metabolism, but no significant regulatory effect was found (**Supplementary Figure S3A**).

HMGCR is a key rate-limiting enzyme in the mevalonate (MVA) pathway that catalyzes the conversion of HMG-CoA to MVA (39–41). Next, MVA can be further transformed into IPP and CoQ10, and these metabolites directly or indirectly promote cell ferroptosis resistance through the GSH/GPX4 axis and FSP1/CoQ10 axis (39–41). As shown, erastin inhibited the growth of NPC cells, and its inhibitory effect was attenuated by ferrostatin-1 (**Supplementary Figure S4A**). Knockdown of HMGCR alleviated ferroptosis resistance in ECM-detached NPC cells, while ectopic expression of HMGCR promoted ferroptosis resistance in cells (**Supplementary Figure S4A**). Moreover, the addition of MVA, whose production is catalyzed by HMGCR, reversed the regulatory effect of CAPRIN2 on NPC cell ferroptosis (**Supplementary Figure S3B**).

### The CAPRIN2/HMGCR axis promotes the ferroptosis resistance and survival of ECM-detached NPC cells

**Figure 2:**
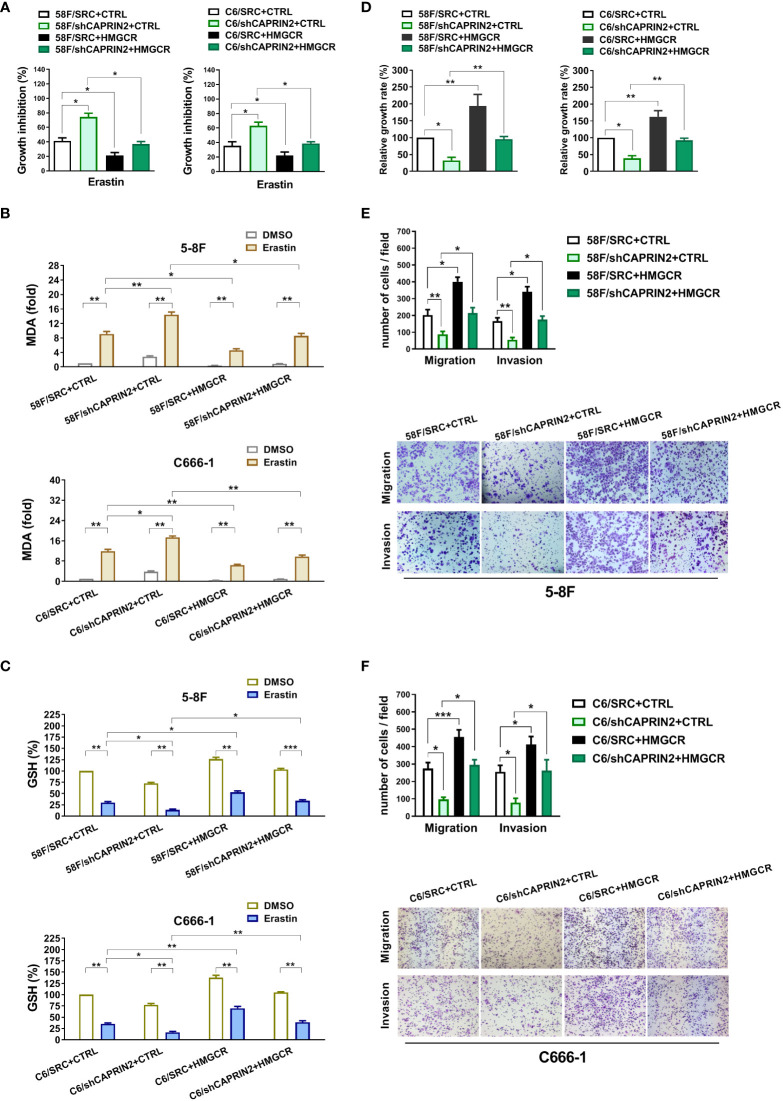
CAPRIN2 promotes the ferroptosis resistance, survival, migration and invasion of NPC cells through HMGCR. **(A)** Overexpression of HMGCR partially reverses the effects of CAPRIN2 on the ferroptosis of ECM-detached 5-8F (left panel) and C666-1 (right panel) cells. The NPC cell lines were treated with erastin (5 μM) for 24 h **(B, C)** MDA assay **(B)** and GSH assay **(C)** results of erastin-treated NPC stable cell lines as indicated. **(D)** Ectopic expression of HMGCR partially rescues the effects of CAPRIN2 knockdown on ECM-detached NPC cell survival. For **(A–C)** and **(D)**, the experiments were conducted in triplicate, and the data are presented as the mean ± SEM. * p < 0.05, ** p < 0.01. **(E, F)** Stable overexpression of HMGCR partially reverses the effects of CAPRIN2 knockdown on 5-8F **(E)** and C666-1 **(F)** cell migration and invasion. Representative images of three independent experiments are shown. The data are expressed as the mean ± SD. * p < 0.05, ** p < 0.01, *** p < 0.001.

To investigate whether HMGCR was involved in the regulation of CAPRIN2 on NPC cell ferroptosis, we constructed CAPRIN2/HMGCR double stable NPC cell lines (**Supplementary Figures S5A, B**). The results indicated that stable overexpression of HMGCR partially reversed the regulatory effects of CAPRIN2 knockdown on ferroptosis resistance (**Figure 2A**). In the indicated erastin-treated cells, we evaluated the level of MDA, a lipid peroxidation product used as a ferroptosis marker. The results showed that HMGCR overexpression partially reversed the increase in MDA levels resulting from CAPRIN2 knockdown (**Figure 2B**). Erastin treatment inhibits cysteine uptake, resulting in decreased GSH synthesis in cells. We measured GSH levels in the indicated NPC stable cell lines after erastin administration. We found that knockdown of CAPRIN2 further enhanced erastin-induced GSH reduction in 5-8F cells, while overexpression of HMGCR partially reversed this effect (**Figure 2C**). Consistent results were also obtained in C666-1 cells (**Figure 2C**).

In addition, we investigated the regulatory effect of the CAPRIN2/HMGCR axis on the survival of ECM-detached NPC cells. Knockdown of HMGCR decreased the survival of ECM-detached NPC cells, while ectopic expression of HMGCR promoted survival (**Supplementary Figure S4B**). As shown in **Figure 2D**, overexpression of HMGCR partially reversed the inhibitory effects of CAPRIN2 knockdown on the survival of ECM-detached cells.

### The effects of CAPRIN2 on promoting metastasis of NPC cells were mediated by HMGCR

Knockdown of HMGCR inhibited the migration and invasion of NPC cells, while overexpression of HMGCR promoted NPC cell migration and invasion (**Supplementary Figures S6A, B**). Moreover, the inhibition of migration or invasion caused by CAPRIN2 knockdown was partially reversed by HMGCR overexpression in 5-8F stable cell lines (**Figure 2E**). Similar results were obtained in C666-1 stable cell lines (**Figure 2F**).

### CAPRIN2 promotes the lung colonization of NPC cells through HMGCR

**Figure 3:**
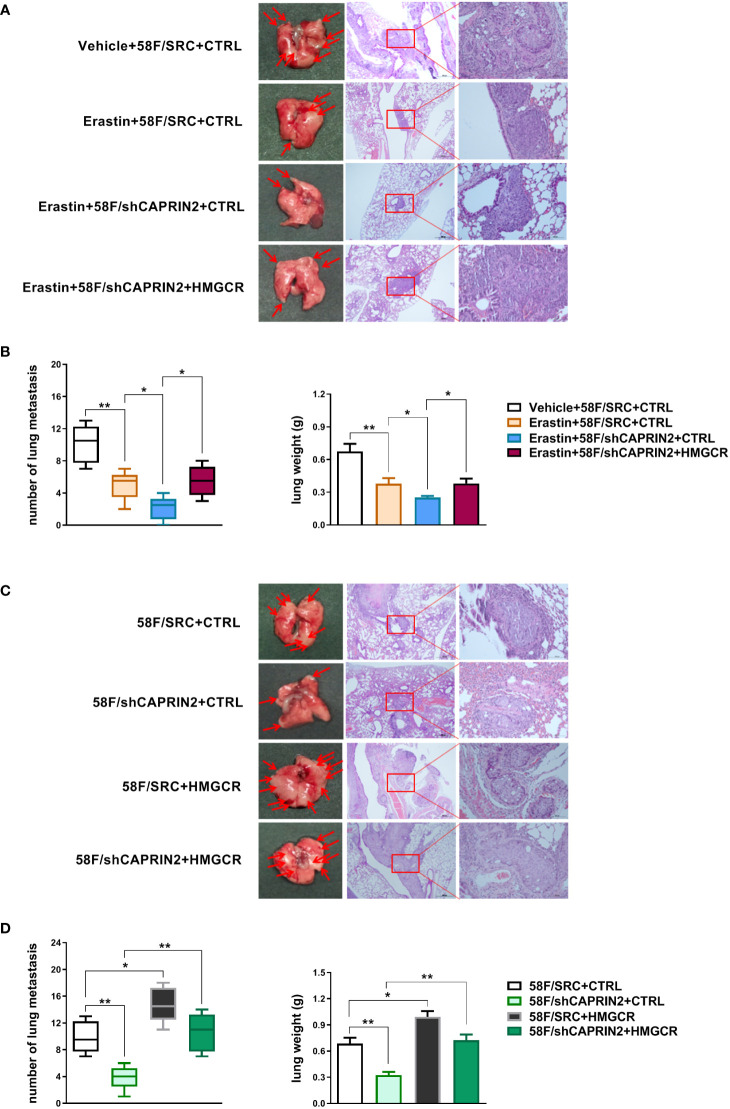
Regulation of lung colonization capacity *via* the CAPRIN2/HMGCR axis in NPC cells. **(A, B)** The inhibitory effect of erastin on the lung metastasis of NPC cells was enhanced by knockdown of the CAPRIN2/HMGCR axis. **(C, D)** CAPRIN2 promotes the lung colonization of NPC cells through HMGCR. For **(A, C)**, representative images of lungs and HE staining are shown. The location of lung metastatic nodules is indicated by the arrow. For **(B, D)**, the number of lung metastases (left panel) and the weight of the lungs (right panel) are given. * p < 0.05, ** p < 0.01.

We first examined the effect of erastin, a ferroptosis inducer, on the lung colonization of NPC cells. The results showed that erastin significantly inhibited the lung metastasis of NPC cells (**Figures 3A, B** **)**. Knockdown of CAPRIN2 promoted the antimetastatic effect of erastin, which was partially reversed by overexpression of HMGCR (**Figures 3A, B** **)**.

ECM detachment alone is enough to be an important trigger for ferroptosis. Tumor cells detached from primary foci must survive ECM detachment stress in blood vessels to reach distal organs and eventually form metastases. Our results showed that knockdown of CAPRIN2 significantly reduced the lung metastasis ability of NPC cells injected through the tail vein, while overexpression of HMGCR partially reversed this effect (**Figures 3C, D** **)**.

### LINC00941 induces CAPRIN2 expression, thereby protecting NPC cells from ferroptosis, maintaining cell survival and promoting metastasis

It has been reported that CAPRIN2 is activated by LINC00941 through DNA looping in OSCC, which is involved in promoting cell proliferation and tumor formation (35). At present, it is not clear whether CAPRIN2 is also regulated by LINC00941 in NPC and whether the LINC00941/CAPRIN2 axis is involved in regulating ferroptosis and metastasis of tumor cells.

To investigate whether LINC00941 is the upstream regulator of CAPRIN2 in NPC, LINC00941 was stably knocked down in 5-8F or C666-1 cells (**Supplementary Figures S5C, D**). As shown, downregulation of LINC00941 led to a decrease in CAPRIN2 and HMGCR expression levels in NPC cells (**Supplementary Figures S5C, D**).

**Figure 4:**
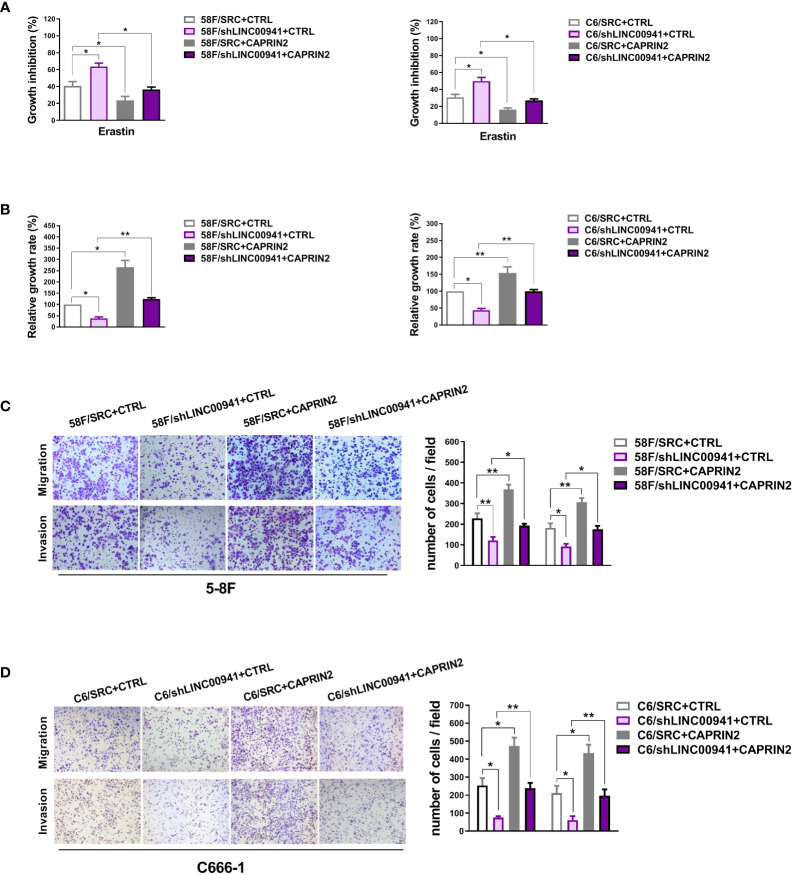
LINC00941 acts as an upstream molecule to regulate the biological functions of CAPRIN2. **(A)** LINC00941 downregulation promoted the ferroptosis of ECM-detached NPC cells, which was partially rescued by CAPRIN2 overexpression. The NPC cells were treated with erastin (5 μM) for 24 h **(B)** Knockdown of LINC00941 decreased the survival of ECM-detached NPC cells, which could be partially reversed by CAPRIN2 overexpression. For **(A)** and **(B)**, the assays were conducted in triplicate, and the data are presented as the mean ± SEM. * p < 0.05, ** p < 0.01. **(C, D)** LINC00941 knockdown inhibited the migration and invasion of 5-8F **(C)** and C666-1 **(D)** cells, and overexpression of CAPRIN2 partially reversed this effect. Representative images are shown. The data are provided as the mean ± SD. * p < 0.05, ** p < 0.01, *** p < 0.001.

Moreover, knockdown of LINC00941 weakened the ferroptosis resistance and survival of ECM-detached NPC cells, while overexpression of CAPRIN2 partially rescued the effects of LINC00941 (**Figures 4A, B** **)**. Downregulation of LINC00941 led to a decrease in the migration and invasion capability of NPC cells, which could be partially reversed by CAPRIN2 overexpression (**Figures 4C, D** **)**.

### CAPRIN2 predicts a poor outcome in NPC patients

**Figure 5:**
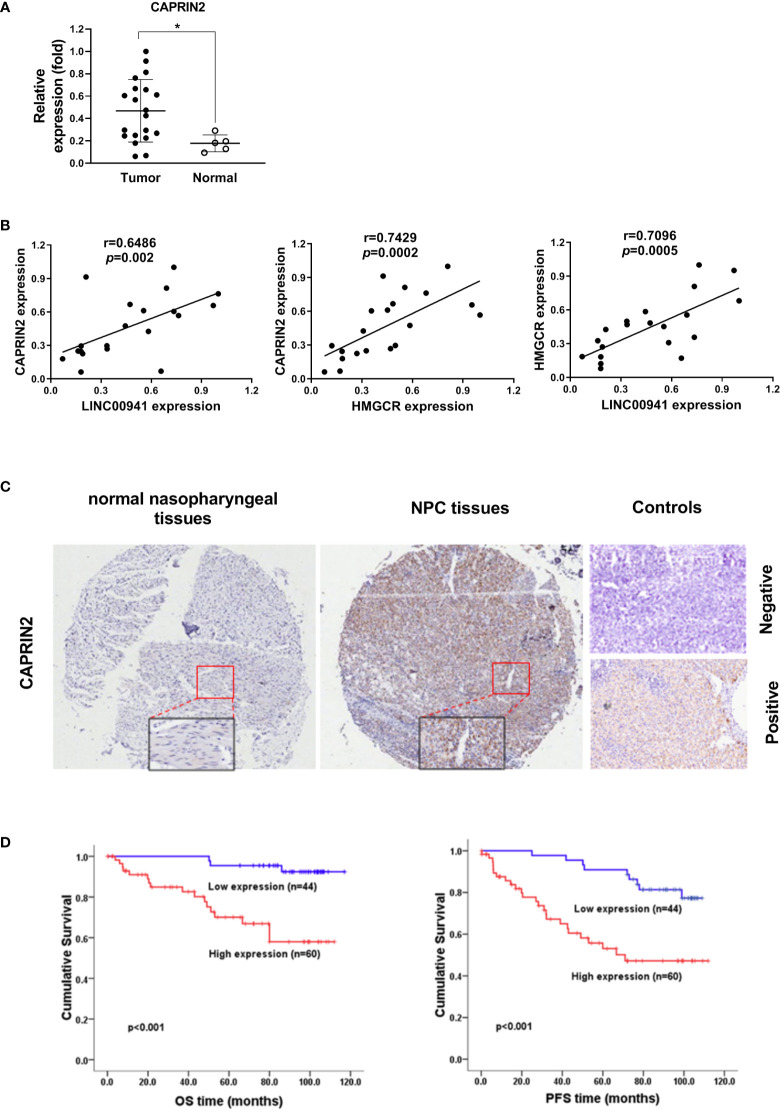
CAPRIN2 is overactivated in NPC tissues and is associated with a poor prognosis in patients. **(A)** The expression level of CAPRIN2 in 20 NPC tissues and 5 nasopharyngeal tissues. **(B)** The correlation between LINC00941/CAPRIN2, CAPRIN2/HMGCR or LINC00941/HMGCR in 20 NPC tissues. For **(A)** and **(B)**, the expression levels of CAPRIN2, HMGCR and LINC00941 were determined by qRT-PCR. The levels were normalized to those of β-actin and shown as the mean ± SEM. * p < 0.05. **(C)** Representative immunohistochemical images of normal nasopharyngeal tissues (left panel) and NPC tissues (middle panel). The boxes represent the magnified region. The representative image of negative stained control (right panel, top) shows the negative staining result of NPC tissues incubated with antibody-free serum. The representative image of positive stained control (right panel, bottom) shows the positive staining result of CAPRIN2 in NPC tissues incubated with the primary antibody of CAPRIN2. **(D)** *Kaplan-Meier* survival analysis of the association between CAPRIN2 expression and the PFS or OS of NPC patients (*log-rank test*).

The expression level of CAPRIN2 was detected in NPC tissues and nasopharynx tissues, and the results showed that CAPRIN2 was highly expressed in NPC tissues (**Figure 5A**). We also examined the pairwise correlations among the expression levels of LINC00941, CAPRIN2, and HMGCR in the above NPC tissues by qRT-PCR. The results indicated that positive correlations between LINC00941 and CAPRIN2, CAPRIN2 and HMGCR, LINC00941 and HMGCR were detected in the above NPC tissues (**Figure 5B**).

To further assess the clinical significance of CAPRIN2 expression in NPC patients, we performed immunohistochemistry and *Kaplan-Meier* analysis. The results showed that CAPRIN2 was overexpressed in NPC tissues, and high expression of CAPRIN2 indicated a shorter progression-free survival (PFS) and overall survival (OS) time than low expression of CAPRIN2 (**Figures 5C, D** **)**. In addition, we also analyzed the association between CAPRIN2 expression and clinical characteristics. The results revealed that there were significant correlations between CAPRIN2 expression and clinicopathologic characteristics, including tumor-node-metastasis (TNM) stage, tumor invasion depth, node metastasis, and distant metastasis (**Table 1**). As shown in **Table 2**, multivariate Cox proportional hazards regression analysis indicated that CAPRIN2 expression acted as an independent prognostic factor for OS in NPC patients.

**Table 1:** Correlations between CAPRIN2 expression and clinicopathological characteristics.

Variables	N (%)	Low CAPRIN2	High CAPRIN2	p values
		expression (%)	expression (%)	
Total cases	104	44 (42.3%)	60 (57.7%)	
Age(years)				
<45	44 (42.3%)	19 (18.3%)	25 (24.0%)	1
≥45	60 (57.7%)	25 (24.0%)	35 (33.7%)	
Sex				
Male	71 (68.3%)	31 (29.8%)	40 (38.5%)	0.831
Female	33 (31.8%)	13 (12.5%)	20 (19.3%)	
TNM stage				
1–2	32 (30.8%)	23 (22.1%)	9 (8.7%)	0
3–4	72 (69.2%)	21 (20.2%)	51 (49.0%)	
T stage				
T1-T2	43 (41.3%)	26 (25.0%)	17 (16.3%)	0.002 ^a^
T3-T4	61 (58.7%)	18 (17.3%)	43 (41.4%)	
N stage				
N<1	30 (28.8%)	20 (19.2%)	10 (9.6%)	0.002 ^a^
N≥1	74 (71.2%)	24 (23.1%)	50 (48.1%)	
Distant metastasis				
No	95 (91.3%)	44 (42.3%)	51 (49.0%)	0.01 ^a^
Yes	9 (8.7%)	0 (0%)	9 (8.7%)	

a Statistically significant.

**Table 2:** Univariate and multivariate analysis for OS.

	Univariate		Multivariate	
Characteristic	hazard ratio (95% CI)	p value	hazard ratio (95% CI)	p value
Age (<45 vs. ≥45)	1.99 (0.723–5.476)	0.183		
TNM stage (I, II vs. III, IV)	5.948 (1.375–25.738)	0.017	2.063 (0.301–14.140)	0.461
Tumor invasion depth (T1–2 vs. T3–4)	2.781 (1.008–7.676)	0.048	1.395 (0.382–5.085)	0.614
Lymph node status (0 vs. ≥1)	1.162 (0.446–3.028)	0.759		
Distant metastasis (no vs. yes)	11.218 (4.230–29.747)	<0.001	5.567 (1.923–16.115)	0.002
CAPRIN2 expression (low vs. high)	7.605 (2.203–26.249)	0.001	4.019 (1.055–15.313)	0.042

## Discussion

Distant metastasis requires the adaptation of tumor cells to the new microenvironment. To successfully form a lung metastatic lesion, ECM-detached tumor cells need to survive in the harsh oxidizing condition of the blood and then adapt to the high oxygen tension in the pulmonary microenvironment (30, 42). To date, little is known about the mechanisms that protect ECM-detached tumor cells from ferroptosis and thus survive. Brown et al. reported that α6β4 integrin promotes resistance to ECM detachment induced ferroptosis (29). Our study showed that CAPRIN2 can inhibit ferroptosis of ECM detached tumor cells and promote cell survival. This is the first time that CAPRIN2 has been reported to play a role in promoting tumor metastasis at the stage of ECM detachment. The high-oxygen lung environment also induces ferroptosis in tumor cells, which is a hindering factor for the formation of lung metastases (30, 42). Alvarez et al. reported that high expression of cysteine desulfurase NFS1 in lung adenocarcinoma protects against oxidative damage in high-oxygen environments (30). Knocking down NFS1 sensitizes cells to glutathione biosynthesis inhibition, which increases ROS and induces tumor cell ferroptosis (30). In our study, the results revealed CAPRIN2 contributes to ferroptosis resistance and lung metastasis loci establishment in NPC cells. In addition, we also found that CAPRIN2 promotes NPC cell migration and invasion. This result is consistent with Zheng *et al*’s report in 2021 that CAPRIN2 can promote the migration and invasion of colorectal cancer cells (43). In conclusion, we believe that CAPRIN2 can be used as a ferroptosis resistance marker and therapeutic target in NPC. Selective inhibition of CAPRIN2 may sensitize NPC cells to oxidative stress and inhibit lung metastasis. In our research on the effects of ECM stiffness on ROS levels, metastasis and ferroptosis of NPC cells (unpublished data), we found that the expression level of CAPRIN2 was upregulated along with increasing ECM stiffness. Whether CAPRIN2 is involved in mediating the effects of ECM stiffness on NPC cell metastasis and ferroptosis remains unknown. In this study, our report on the biological function of CAPRIN2 may provide clues to understand the mechanism by which ECM stiffness affects NPC cell function.

Cells maintained ECM-detached when cultured with ultralow attachment plates, and ECM-attached when cultured with standard cell culture plates. In our preliminary experiments, we also evaluated the effects of CAPRIN2 on proliferation and ferroptosis of NPC cells under ECM-attached conditions. The results showed that knockdown of CAPRIN2 inhibited the proliferation of 5-8F cells at 72h in viability assays and promoted erastin-induced ferroptosis (data not shown), which suggested that CAPRIN2 might also be involved in the malignant phenotype of NPC cells under ECM-attached conditions.

In the process of searching for the mechanism how CAPRIN2 regulates the antioxidant defense molecules involved in cellular ferroptosis resistance, we focused on three antioxidant axes, which mainly involved in regulating cell ROS level and mediating ferroptosis resistance (9). Related core molecules that associated with these regulatory axes were selected for evaluation. For the GSH/GPX4 axis, SLC7A11 (one subunit of the anionic amino acid transport system that is highly specific for cysteine and glutamate), GPX4 (antioxidant selenium enzyme), HMGCR (the key rate-limiting enzyme of MVA pathway) and GCLC (the first rate-limiting enzyme of glutathione synthesis) were evaluated (9). For the FSP1/CoQ10 axis, we examined the level of FSP1, which acts as an independent parallel system to protect cells from ferroptosis (9). For the GCH1/DHFR axis, GCH1 (the rate-limiting enzyme for BH4 synthesis) and dihydrofolate reductase DHFR were detected (9). Addtionally, we also evaluated several key molecules involving in various stages of iron metabolism, including Fe transport (TFRC), Fe storage (FTH1、FTL) and ferritinophagy (NCOA4) (9). The results showed that HMGCR, which mediates ferroptosis resistance through MVA pathway, was activated by CAPRIN2. Therefore, our study uncovered one of the mechanisms by which CAPRIN2 activates the cellular antioxidant defense system in NPC cells.

In addition to being an RNA-binding protein, Caprin2 can also bind to Wnt receptor LRP5/6. The Wnt pathway is one of the carcinogenic pathways that are abnormally activated in NPC. Aberrant activation of this pathway is associated with the promoter methylation of Wnt inhibitors (DKK1, WIF1, SFRP1, SFRP2, SFRP4, and SFRP5) (4). As an LRP5/6-binding protein, Caprin2 is reported to activate the canonical Wnt pathway by regulating LRP5/6 phosphorylation (44). Therefore, the high level of CAPRIN2 may also be involved in the activation of the Wnt pathway in NPC. It has been reported that the Wnt pathway can act as an activator of the MVA pathway (39). Therefore, the positive regulation of HMGCR by CAPRIN2 found in this study might be mediated by the Wnt pathway. It has been reported that products of the MVA pathway can also act as activators to activate the Wnt pathway (41). Therefore, there may be positive feedback regulation between MVA pathway molecules and Wnt pathway molecules.

In summary, we found that CAPRIN2 is a novel regulator of ferroptosis and metastasis in NPC and plays a role through HMGCR, a key enzyme in the MVA pathway. Our study is expected to provide a new marker of ferroptosis resistance and a new therapeutic target for NPC.

## Data availability statement

The original contributions presented in the study are included in the article/**Supplementary Material**. Further inquiries can be directed to the corresponding author.

## Ethics statement

The studies involving human participants were reviewed and approved by SYSUCC Institutional Research Ethics Committee. The patients/participants provided their written informed consent to participate in this study.

The animal study was reviewed and approved by Sun Yat-sen University Institutional Animal Care and Use Committee.

## Author contributions

JW contributed to the concept and design of the study. LQ, RZ, LZ, and SY performed the experiments. LQ, SY, and JW contributed to data analysis and interpretation. JW wrote the manuscript. All authors contributed to the article and approved the submitted version.

## Funding

This work was supported by the National Natural Science Foundation of China (No. 81772885).

## Conflict of interest

The authors declare that the research was conducted in the absence of any commercial or financial relationships that could be construed as a potential conflict of interest.

## Publisher’s note

All claims expressed in this article are solely those of the authors and do not necessarily represent those of their affiliated organizations, or those of the publisher, the editors and the reviewers. Any product that may be evaluated in this article, or claim that may be made by its manufacturer, is not guaranteed or endorsed by the publisher.
