# Supplementary material for: CAPRIN2 upregulation by LINC00941 promotes nasopharyngeal carcinoma ferroptosis resistance and metastatic colonization through HMGCR
Source: Front Oncol. 2022 Oct 6;12:931749. doi: 10.3389/fonc.2022.931749 (PMC9582274; doi:10.3389/fonc.2022.931749)
Supplement: Supplementary file 1 [file DataSheet_1.pdf]

Table S1. qRT-PCR primer sequences of the detected genes and LINC00941.

Primer	Sequence (5'-3')
CAPRIN2 Forward	TGCTGCCCAGGAAGTTGA
CAPRIN2 Reverse	TGAAGGCGGTTGTGACGT
AIFM2 Forward	GTGAGCGGGTGAGCAATCT
AIFM2 Reverse	CTTGATGCCGGTGCAGAGAA
DHFR Forward	TCGCTAAACTGCATCGTCGCTGTGTC
DHFR Reverse	TGGAGGTTCCTTGAGTTCTCTGCTGA
FDFT1 Forward	GCAACGCAGTGTGCATATTTT
FDFT1 Reverse	CGCCAGTCTGGTTGGTAAAGG
FTH1 Forward	GGGAACATGCTGAGAAACTG
FTH1 Reverse	CAGTCTGGTTTCTTGATATCCTG
FTL Forward	CAGCCTGGTCAATTTGTACCT
FTL Reverse	GCCAATTCGCGGAAGAAGTG
GCH1 Forward	GTGAGCATCACTTGGTTCCAT
GCH1 Reverse	GTAAGGCGCTCCTGAACTTGT
GCLC Forward	AACCCAAACCATCCTACCC
GCLC Reverse	ACATTGTTCCTCCGTAGGG
GPX4 Forward	GAGGCAAGACCGAAGTAAACTAC
GPX4 Reverse	CCGAACTGGTTACACGGGAA
HMGCR Forward	TGATTGACCTTTCCAGAGCAAG
HMGCR Reverse	CTAAAATTGCCATTCCACGAGC
NCOA4 Forward	GAGGTGTAGTGATGCACGGAG
NCOA4 Reverse	GACGGCTTATGCAACTGTGAA
SLC7A11 Forward	GCTAATTAAAGGTCAAACGCAG
SLC7A11 Reverse	CCAGCATATGCATACATTCCA
TFRC Forward	ACCATTGTCATATACCCGGTTCA
TFRC Reverse	CAATAGCCCAAGTAGCCAATCAT
LINC00941 Forward	ACCACTACACTCAGCCAAATAC
LINC00941 Reverse	GGCTATCAACTGTCTCCTTTAGAC
β-actin Forward	TGGCACCCAGCACAATGAA
β-actin Reverse	CTAAGTCATAGTCCGCCTAGAAGCA

Supplemental figure legends

Figure S1. Endogenous expression levels of CAPRIN2, HMGCR and LINC00941 in human NPC cell lines (5-8F, 6-10B, HK1 and C666-1) and the NP69 normal nasopharyngeal epithelial cell line. (A) The expression levels of the indicated genes or lncRNAs were quantified by qRT-PCR. β-Actin was used as a loading control. * $p < 0.05$, ** $p < 0.01$. (B) The protein levels of CAPRIN2 and HMGCR were determined by Western blotting. β-Actin was used as an internal control.

Figure S2. Expression levels of CAPRIN2 and HMGCR in the indicated stable cell lines. A-B, CAPRIN2 and HMGCR levels in 5-8F (A) or C666-1 (B) cells with stable CAPRIN2 overexpression or knockdown. The expression levels of mRNA were determined by qRT-PCR. β-Actin was used as the internal control. The data are provided as the mean±SEM. * $p < 0.05$, ** $p < 0.01$, *** $p < 0.001$. The expression levels of proteins were evaluated by Western blotting. β-Actin was used as the loading control.

Figure S3. Potential downstream effectors regulated by CAPRIN2. (A) The expression levels of the indicated genes in 5-8F (upper panel) and C666-1 (lower panel) cells were determined by qRT-PCR. β-Actin was used as an internal control. The data are presented as the mean±SEM. ** $p < 0.01$, *** $p < 0.001$. (B) Viability assay of NPC cells with stable knockdown of CAPRIN2 treated with erastin (5 μM) and/or MVA (100 μM) for 24 h. The results of three repeated experiments are shown as the mean±SEM. * $p < 0.05$, ** $p < 0.01$.

Figure S4. The regulation of ferroptosis and survival by HMGCR in NPC cells. (A, B) NPC cells were transiently transfected with the indicated siRNA or plasmid for 48 h. Afterward, the cells were suspended and cultured with ultralow attachment plates. For (A), cells were given erastin (5 μM) and/or ferrostatin-1 (1 μM) for 24 h. For (B), cell viability was assessed with the Alamer Blue Assay after the 72 h of culture. The assays were conducted in triplicate, and the data are presented as the mean±SEM. * $p < 0.05$, ** $p < 0.01$.

Figure S5. Expression levels of CAPRIN2, HMGCR and LINC00941 in the indicated stable cell lines. (A, B) CAPRIN2 and HMGCR levels in 5-8F (A) and C666-1 (B) cells with stable knockdown of CAPRIN2 and/or stable overexpression of HMGCR. (C, D) CAPRIN2, HMGCR and LINC00941 levels in 5-8F (C) and C666-1 (D) cells with stable knockdown of LINC00941 and/or stable overexpression of CAPRIN2. The expression levels of the indicated mRNAs or lncRNA were determined by qRT-PCR. β-Actin was used as an internal control. The data are shown as the mean±SEM. ** $p < 0.01$, *** $p < 0.001$; n.s., not significant. The expression levels of proteins were evaluated by Western blotting. β-Actin was used as the loading control.

Figure S6. The regulation of NPC cell migration and invasion by HMGCR. (A, B) Migration and invasion assays of 5-8F (A) and C666-1 (B) cells transiently transfected with the indicated siRNAs (upper panel) or plasmids (lower panel). Representative images are shown. The experiment was repeated three times, and the data are given as the mean±SD. * $p < 0.05$, ** $p < 0.01$, *** $p < 0.001$.

S Fig.1

A

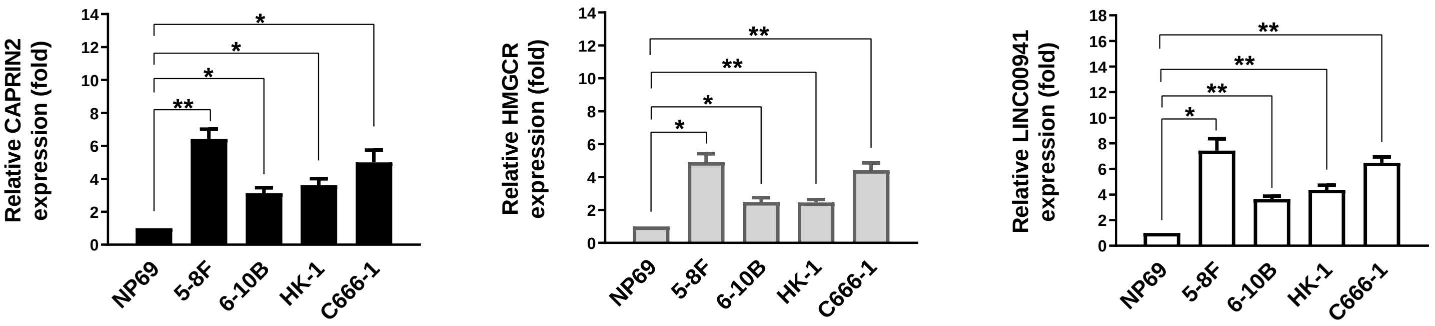

Relative CAPRIN2 expression (fold)
Relative HMGCR expression (fold)
Relative LINC00941 expression (fold)
NP69
5-8F
6-10B
HK-1
C666-1
*
**

B

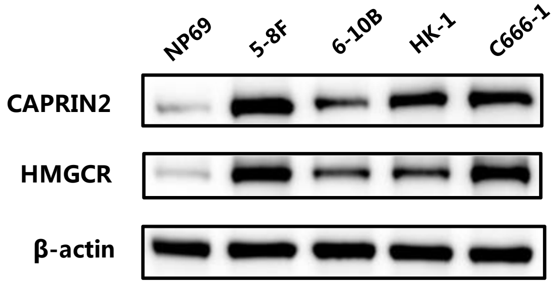

NP69
5-8F
6-10B
HK-1
C666-1
CAPRIN2
HMGCR
β-actin

S Fig.2

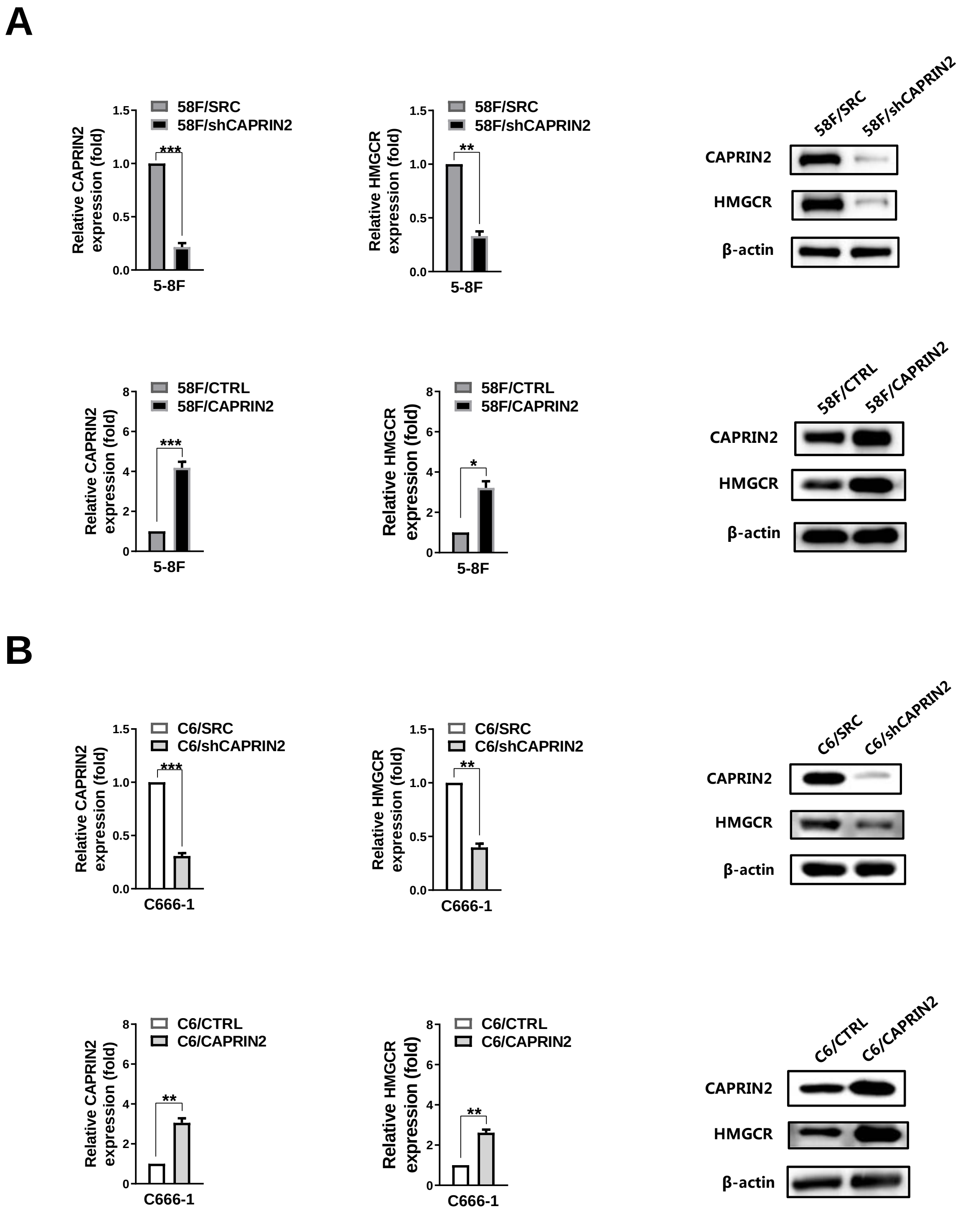
A
58F/SRC
58F/shCAPRIN2
Relative CAPRIN2 expression (fold)
1.5
1.0
0.5
0.0
***
5-8F
Relative HMGCR expression (fold)
**
58F/SRC
58F/shCAPRIN2
CAPRIN2
HMGCR
β-actin
58F/CTRL
58F/CAPRIN2
8
6
4
2
0
***
*
58F/CTRL
58F/CAPRIN2
CAPRIN2
HMGCR
β-actin
B
C6/SRC
C6/shCAPRIN2
***
**
C666-1
C6/SRC
C6/shCAPRIN2
CAPRIN2
HMGCR
β-actin
C6/CTRL
C6/CAPRIN2
**
**
C666-1
C6/CTRL
C6/CAPRIN2
CAPRIN2
HMGCR
β-actin

S Fig.3

A

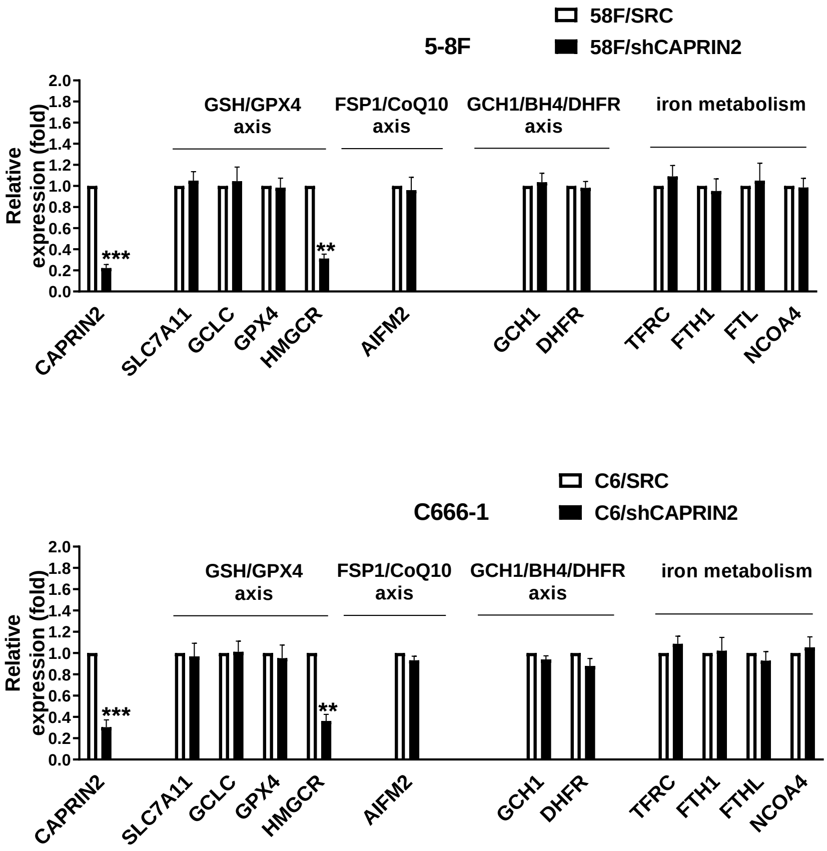
5-8F
58F/SRC
58F/shCAPRIN2
Relative expression (fold)
2.0
1.8
1.6
1.4
1.2
1.0
0.8
0.6
0.4
0.2
0.0
GSH/GPX4 axis
FSP1/CoQ10 axis
GCH1/BH4/DHFR axis
iron metabolism
***
**
CAPRIN2
SLC7A11
GCLC
GPX4
HMGCR
AIFM2
GCH1
DHFR
TFRC
FTH1
FTL
NCOA4
C666-1
C6/SRC
C6/shCAPRIN2
Relative expression (fold)
2.0
1.8
1.6
1.4
1.2
1.0
0.8
0.6
0.4
0.2
0.0
GSH/GPX4 axis
FSP1/CoQ10 axis
GCH1/BH4/DHFR axis
iron metabolism
***
**
CAPRIN2
SLC7A11
GCLC
GPX4
HMGCR
AIFM2
GCH1
DHFR
TFRC
FTH1
FTHL
NCOA4

B

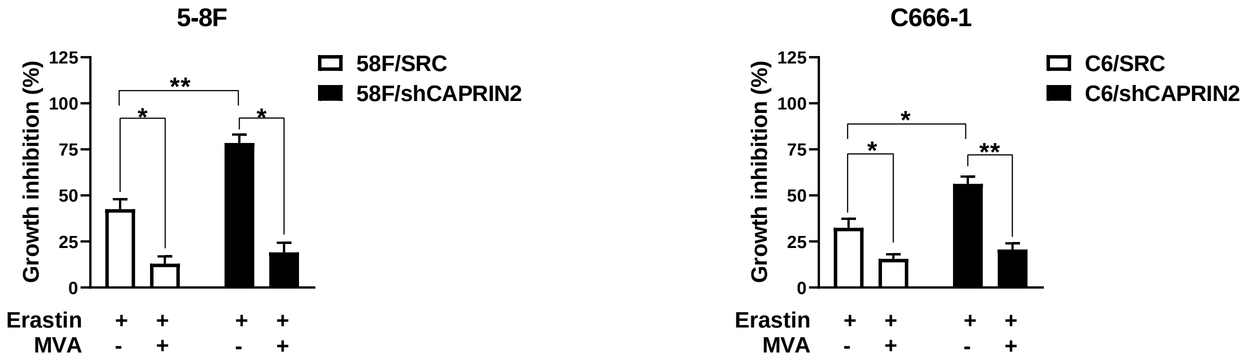
5-8F
58F/SRC
58F/shCAPRIN2
Growth inhibition (%)
125
100
75
50
25
0
**
*
*
Erastin + + + +
MVA - + - +
C666-1
C6/SRC
C6/shCAPRIN2
Growth inhibition (%)
125
100
75
50
25
0
*
*
**
Erastin + + + +
MVA - + - +

S Fig.4

A

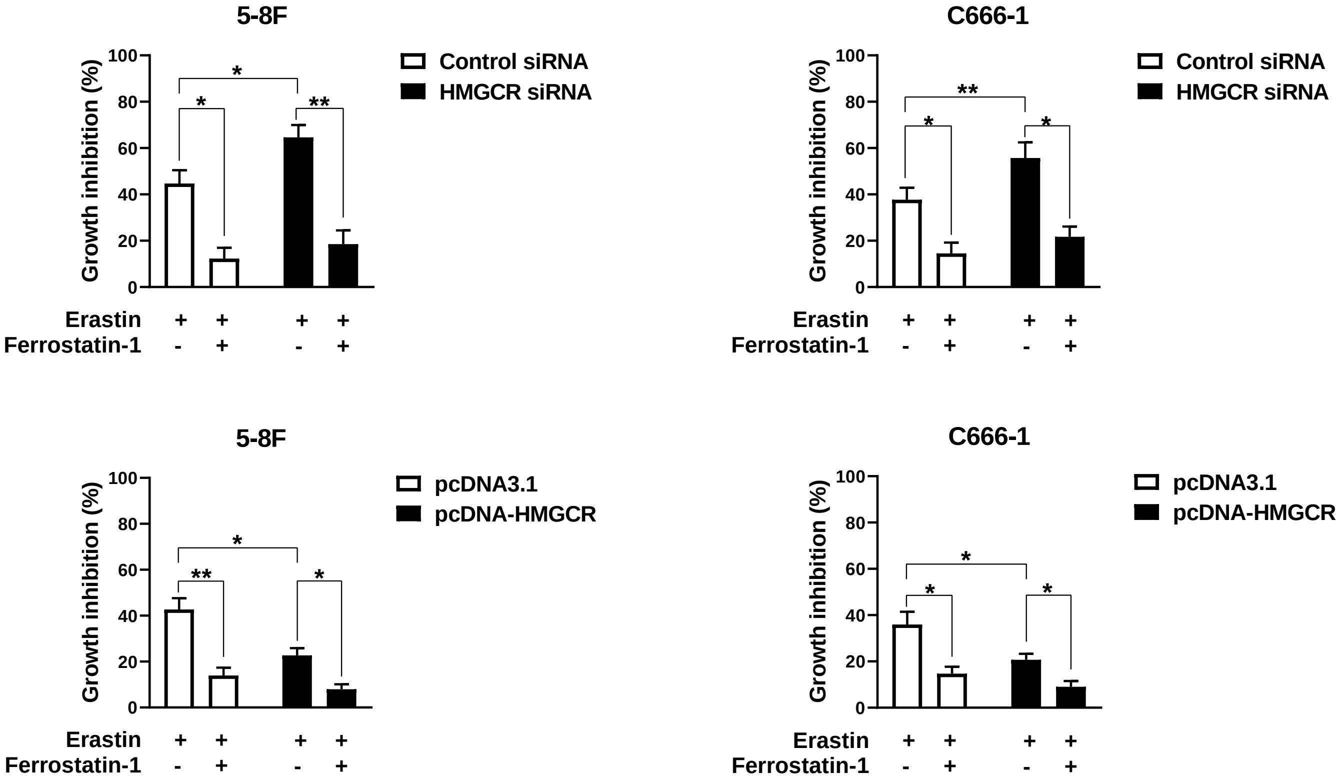
5-8F
C666-1
Growth inhibition (%)
Control siRNA
HMGCR siRNA
pcDNA3.1
pcDNA-HMGCR
Erastin
Ferrostatin-1
0
20
40
60
80
100
*
**

B

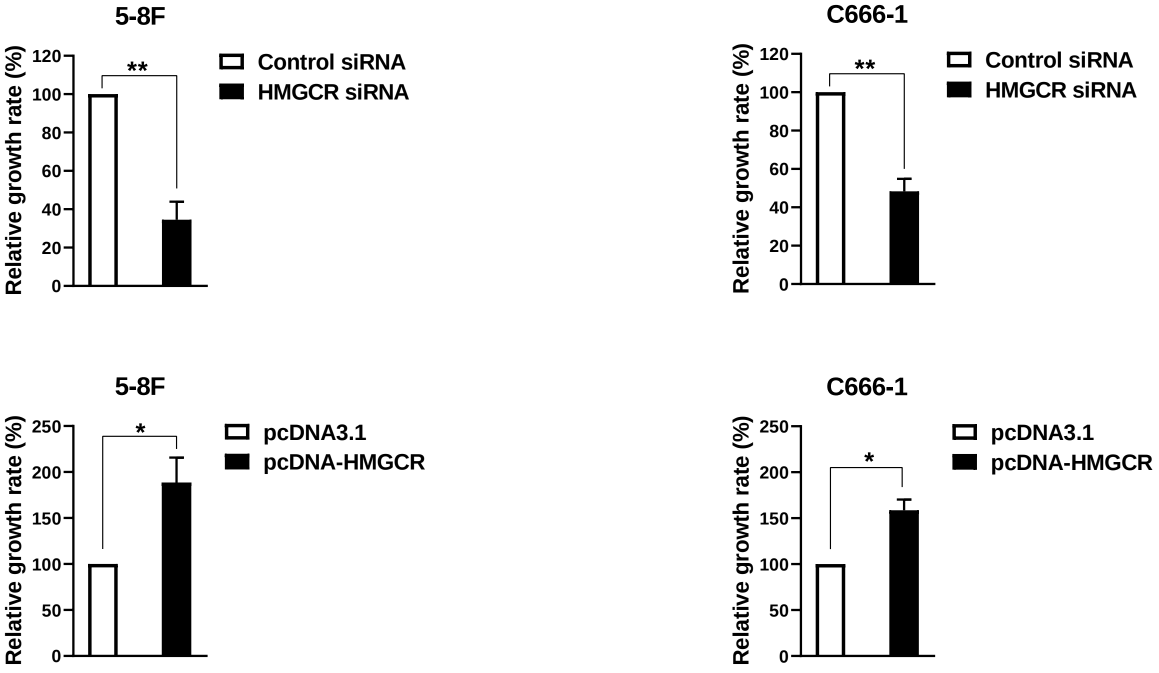
5-8F
C666-1
Relative growth rate (%)
Control siRNA
HMGCR siRNA
pcDNA3.1
pcDNA-HMGCR
0
20
40
60
80
100
120
50
150
200
250
*
**

S Fig.5

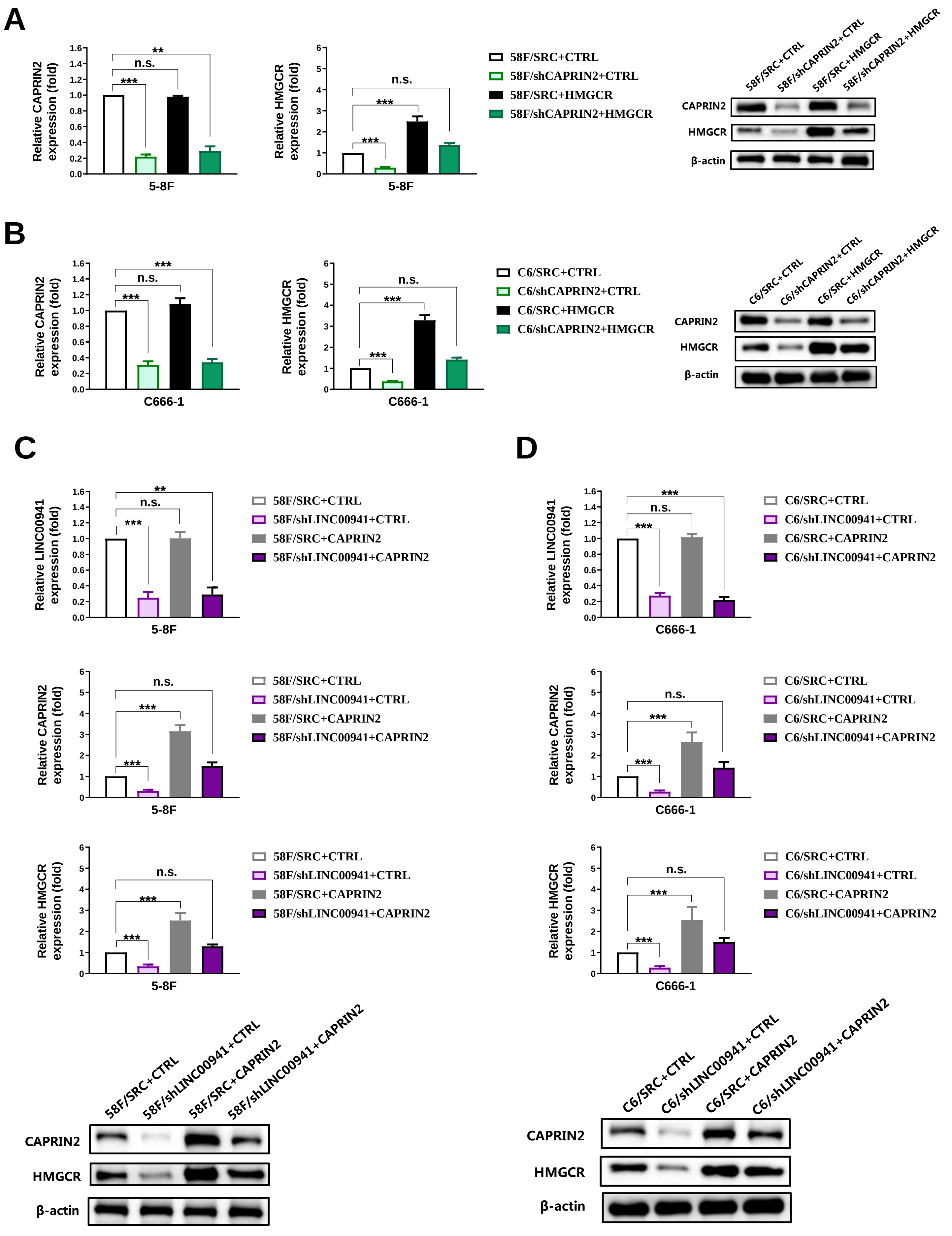
A
Relative CAPRIN2 expression (fold)
**
n.s.
***
5-8F
Relative HMGCR expression (fold)
n.s.
***
***
5-8F
58F/SRC+CTRL
58F/shCAPRIN2+CTRL
58F/SRC+HMGCR
58F/shCAPRIN2+HMGCR
CAPRIN2
HMGCR
β-actin
B
Relative CAPRIN2 expression (fold)
***
n.s.
***
C666-1
Relative HMGCR expression (fold)
n.s.
***
***
C666-1
C6/SRC+CTRL
C6/shCAPRIN2+CTRL
C6/SRC+HMGCR
C6/shCAPRIN2+HMGCR
CAPRIN2
HMGCR
β-actin
C
Relative LINC00941 expression (fold)
**
n.s.
***
5-8F
58F/SRC+CTRL
58F/shLINC00941+CTRL
58F/SRC+CAPRIN2
58F/shLINC00941+CAPRIN2
Relative CAPRIN2 expression (fold)
n.s.
***
***
5-8F
Relative HMGCR expression (fold)
n.s.
***
***
5-8F
CAPRIN2
HMGCR
β-actin
D
Relative LINC00941 expression (fold)
***
n.s.
***
C666-1
C6/SRC+CTRL
C6/shLINC00941+CTRL
C6/SRC+CAPRIN2
C6/shLINC00941+CAPRIN2
Relative CAPRIN2 expression (fold)
n.s.
***
***
C666-1
Relative HMGCR expression (fold)
n.s.
***
***
C666-1
CAPRIN2
HMGCR
β-actin

S Fig.6

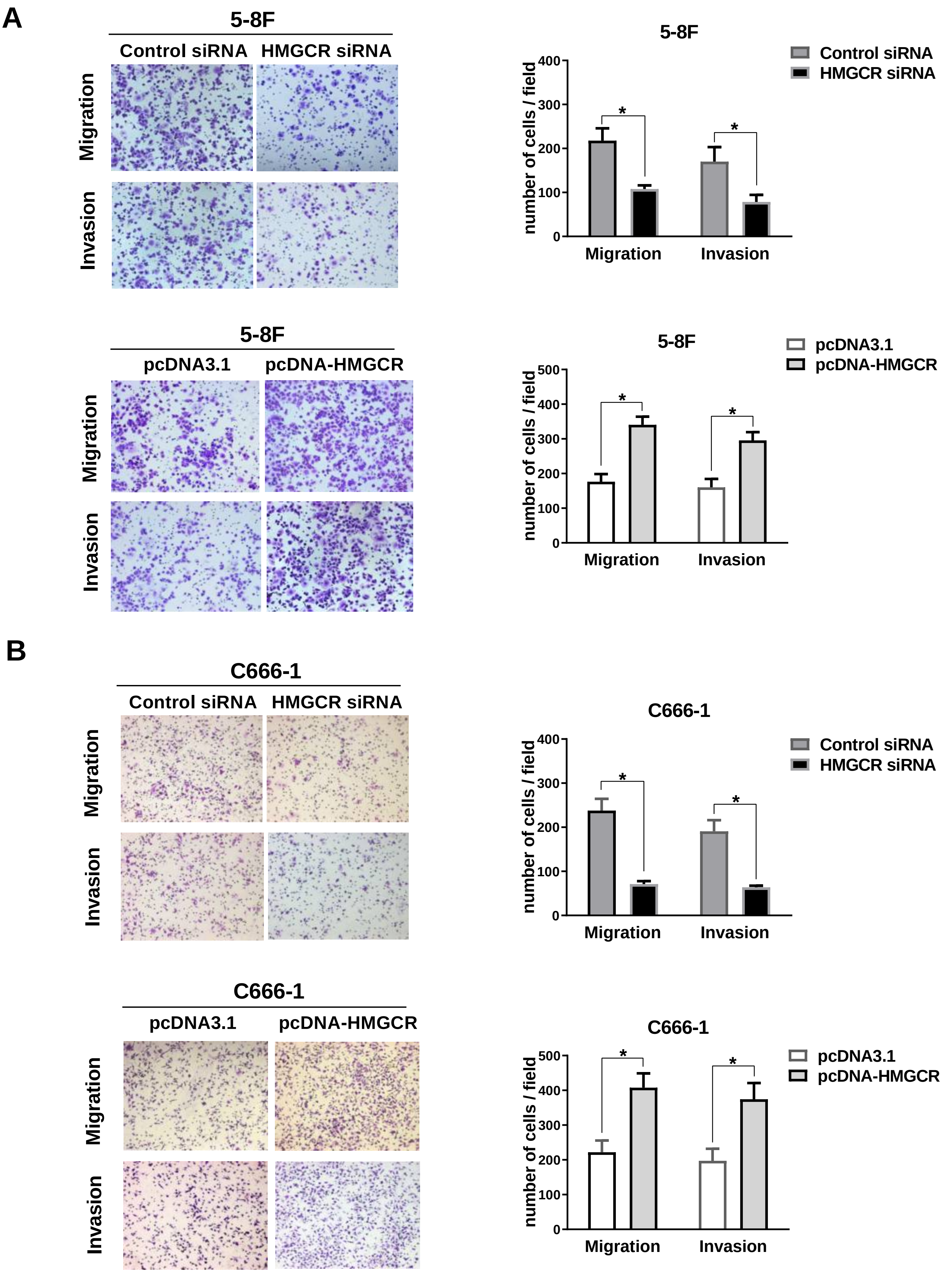
A
5-8F
Control siRNA
HMGCR siRNA
Migration
Invasion
5-8F
Control siRNA
HMGCR siRNA
number of cells / field
0
100
200
300
400
*
*
Migration
Invasion
5-8F
pcDNA3.1
pcDNA-HMGCR
Migration
Invasion
5-8F
pcDNA3.1
pcDNA-HMGCR
number of cells / field
0
100
200
300
400
500
*
*
Migration
Invasion
B
C666-1
Control siRNA
HMGCR siRNA
Migration
Invasion
C666-1
Control siRNA
HMGCR siRNA
number of cells / field
0
100
200
300
400
*
*
Migration
Invasion
C666-1
pcDNA3.1
pcDNA-HMGCR
Migration
Invasion
C666-1
pcDNA3.1
pcDNA-HMGCR
number of cells / field
0
100
200
300
400
500
*
*
Migration
Invasion
